# High Risk Pregnancy and its Outcome in a Tertiary Care Hospital: A Descriptive Cross-sectional Study

**DOI:** 10.31729/jnma.8561

**Published:** 2024-05-31

**Authors:** Sunita Bhandari, Yam Dwa, Meenu Maharjan, Manisha Bajracharya

**Affiliations:** 1 Department of Obstetrics and Gynecology, KIST Medical College and Teaching Hospital, Lalitpur, Nepal

**Keywords:** *high risk pregnancy*, *risk factors*, *pregnancy outcome*

## Abstract

**Introduction:**

High-risk pregnancy is defined as one which is complicated by factors or factors that adversely affect the pregnancy outcome (maternal, perinatal or both). Early detection and effective management of high risk pregnancy helps in achieving favorable maternal and perinatal outcomes. This study aimed to find the prevalence of high risk pregnancy and its outcome among pregnant women admitted for delivery in the obstetrics and gynecology department of a tertiary care hospital.

**Methods:**

A descriptive cross sectional study was conducted in a tertiary care hospital among high risk pregnant women admitted for delivery using structured proforma, from April 2023 to September 2023 after obtaining ethical approval from the Institutional Review Committee. Convenience sampling was used among pregnant women who met the inclusion criteria. Data was entered in excel and analysis was done using IBM SPSS software. Point estimate was calculated at 95% Confidence Interval.

**Results:**

Among 350 deliveries, high risk pregnancy was seen in 91 (26%) (16.15-32.00, 95% Confidence Interval). The high risk factors were previous history of cesarean section 25 (27.47 %) followed by hypothyroidism 19 (20.87%) and gestational diabetes mellitus 15 (16.48%). Out of 91 high risk pregnancy, 84 (92.30%) had term delivery. Lower segment cesarean section was done in 69 (75.82%) patients of which 26 (28.57%) underwent emergency cesarean section. The total number of births among high risk preganancies were 93 with two sets of twin births. A total of 13 (13.97%) of the babies had low-birth weight.

**Conclusions:**

The prevalence of high risk pregnancy was found to be similar as compared to studies done in similar settings.

## INTRODUCTION

High-risk pregnancy (HRP) is defined as one which is complicated by factors that adversely affect the pregnancy outcome (maternal, perinatal or both). Every day in 2020, almost 800 women died from preventable causes related to pregnancy and childbirth, according to WHO.^1^ About 20-30% of all pregnancies belong to high risk which is responsible for 70-80% of perinatal mortality and morbidity.^2^

It is essential to study about the high risk factors associated with pregnancy for early detection and timely management so as to ensure the best possible maternal,obstetric, and neonatal outcome.

This study was conducted to determine the prevalence of high risk pregnancy and its outcome among pregnant women admitted for delivery in the obstetrics and gynecology department of a tertiary care hospital.

## METHODS

A descriptive cross-sectional study was conducted among high risk pregnant women admitted for delivery in the department of Obstetrics and Gynecology in KIST Medical College and Teaching Hospital (KISTMCTH), Lalitpur, Nepal after obtaining ethical approval from the Institutional Review Committee of the same institute (Reference number: 2079/80/116). Patients were informed regarding the study and informed written consent was taken prior to the study. The women who met the inclusion criteria were recruited consecutively from 1^st^ April 2023 to 30^th^ September 2023.

The study included all pregnant women from gestational age of 28 weeks and above admitted for delivery with at least one high risk factor. A convenience sampling method was used. The sample size was calculated using the following formula:


$$\begin{array}{ll} \text{n} & ={\text{Z}}^{2}\times \frac{\text{p}\times \text{q}}{{\text{e}}^{\text{2}}}\\ & =1.96^{2}\times \frac{0.1581\times 0.8419}{0.05^{2}}\\ & =205 \end{array}$$

Where,

n= minimum required sample size
Z= 1.96 at 95% Confidence Interval (CI)
p= proportion of condition in population taken (15.81%) according to previous study^5^
q= 1-p
e= margin of error, 5%

The calculated sample size was 205. However, 350 women were enrolled during the study period. A structured proforma comprising socio-demographic variables, obstetric variables, and associated risk factors of high risk pregnancy were filled after taking detailed history from each pregnant woman and they were followed up till delivery. Obstetric and neonatal outcomes were noted.

Women with the following conditions were categorized under high-risk pregnancy: severe anemia with hemoglobin level <7 g/dl, hypertensive disorder in pregnancy (blood pressure >140/90 mm of Hg), pregnant women positive for HIV/syphilis, hypothyroidism (thyroid-stimulating hormone level above the normal values - first trimester: 0.1-2.5 mIU/L, second trimester: 0.2-3 mIU/L, and third trimester: 0.3-3 mIU/L), gestational diabetes mellitus (glucose challenge test ≥140 mg/dl), twin pregnancy or multiple pregnancy, previous history of lower segment cesarean section, younger primi (age <20 years) or elderly primi (age >35 years), malpresentation, bad obstetric history (history of congenital malformation, stillbirth, abortion, premature birth, and obstructed labor), Rh incompatibility, low-lying placenta or placenta previa.^2^

High risk pregnant women admitted for other problem and not planned for immediate delivery were excluded in the study.

Data was entered in excel and analysis was done using IBM SPSS. The point estimate was calculated at a 95% confidence interval. Frequency and percentage analysis was done for the categorical variables.

## RESULTS

The number of deliveries during the study period was 350. Out of 350 deliveries, the prevalence of high risk pregnancy was 91 (26%) (16.15-32.00, 95% Confidence Interval). Majority 136 (38.85%) of pregnant women were in the age group 26-30 years. Most of the women were literate with 209 (59.71%) having a secondary level of education. Around 241 (68.85%) women were involved in house work. Around 333 (95.14%) of study participants belong to the middle socioeconomic class (Table 1).

**Table 1:** Sociodemographic variables of study population (n= 350).

Variables	n (%)
**Age in years**	
<20	4 (1.14)
20-25	103 (29.42)
26-30	136 (38.85)
31-35	77 (22)
36-40	30 (8.57)
**Residence**	
Urban	265 (75.71)
Rural	85 (24.28)
**Educational level**	
Illiterate	11 (3.14)
Primary	99 (28.28)
Secondary	209 (59.71)
University	31 (8.85)
**Occupation**	
Formal employment	17 (4.85)
Self-employment	74 (21.14)
Student	18 (5.14)
House wife	241 (68.85)
**Socioeconomic status**	
Low	3 (0.85)
Middle	333 (95.14)
High	14 (4)

Out of 91 (26%) high risk pregnant women, 84 (92.30%) were term pregnancy from 37-42 weeks, while 6 (6.59%) were preterm and 1 (1.09%) was post term pregnancy. There were 29 (31.86%) primi and 62 (68.13%) multi gravida. There were 75 (82.41%) booked cases with ≥4 antenatal care (ANC) visits 84 (92.30%) (Table 2).

**Table 2:** Obstetrical variables of High risk pregnant women (n= 91).

Variables	n (%)
**Gestational age**	
28-36 weeks+ 6 day	6 (6.59)
37-42 weeks	84 (92.30)
>42 weeks	1 (1.09)
**Gravida**	
Primi	29 (31.86)
Multi	62 (68.14)
**Parity**	
0	29 (31.86)
1	48 (52.76)
2	11 (12.09)
3	2 (2.19)
≥4	1 (1.09)
**Number of living child**	
0	37 (40.67)
1	46 (50.56)
2	5 (5.49)
3	2 (2.19)
4	1 (1.09)
**Previous abortion**	
0	84 (92.30)
1	2 (26.38)
2	3 (3.29)
3 or more	2 (2.19)
**Antenatal care**	
Booked	75 (82.42)
Unbooked	16 (17.58)
**ANC visits**	
<4	7 (7.70)
≥4	84 (92.30)

Among these high risk pregnant women, 76 (83.51%) had a single high risk factor, and the remaining 15 (16.48%) had more than one high risk factor. Among 91 (26%) high risk pregnant women, the previous history of lower secgment cesarean section (LSCS) was 25 (27.47 %) followed by hypothyroidism 19 (20.87%), and gestational diabetes mellitus 15 (16.48%) (Table 3).

**Table 3:** Risk factors of high risk pregnancy (n= 91).

Variables	n (%)
Previous history of LSCS	25 (27.47)
Hypothyroidism	19 (20.87)
Gestational diabetes mellitus	15 (16.48)
Malpresentation	12 (13.17)
Gestational hypertension	7 (7.69)
**Age**	
Younger primi	4 (4.39)
Elderly primi	2 (2.19)
**Bad Obstetric History**	
Congenital Malformation	1 (1.09)
Stillbirth	2 (2.19)
Recurrent Abortion	2 (2.19)
Premature Birth	1 (1.09)
Obstructed Labor	-
Multiple / Twin Pregnancy	2 (2.19)
Severe Anemia	1 (1.09)
**Infection**	
HIV	-
Syphilis	1 (1.09)
Rh incompatibility	1 (1.09)
Placenta previa	1 (1.09)

Majority 84 (92.30%) had term delivery. There were 69 (75.82%) lower segment cesarean section. Only 22 (24.18%) had vaginal delivery (Table 4).

**Table 4:** Obstetric outcome of High risk pregnancy (n= 91).

Variables	n (%)
**Type of Delivery**	
Preterm	6 (6.59)
Term	84 (92.30)
Postterm delivery	1 (1.09)
**Mode of delivery**	
Vaginal delivery	22 (24.18)
Instrumental delivery	-
Elective LSCS	43 (47.25)
Emergency LSCS	26 (28.57)

The total number of births was 93 with two sets of twin births and all 93 (100%) were live births. 13 (13.97%) of the babies had low-birth weight (Table 5).

**Table 5:** Neonatal outcome of High risk pregnancy (n= 93).

Variables	n (%)
**Birth weight of child**	
Low-birth weight baby	13 (13.97)
Normal baby	80 (86.02)
**Status of Birth**	
Live birth	93 (100)
Stillbirth	-
Early neonatal death	-

## DISCUSSION

The prevalence of high risk pregnancy among 350 deliveries during the study period was 91 (26%) in our study which is consistent with previous studies done in Hyderabad, India (2020) and South Western Ethiopia (2021) where prevalence of high risk pregnancy was 25.9% and 26.43% respectively.^3,4^ This study is not in agreement with the studies done in Nepal at Shree Birendra military hospital (2017),and Koshi Hospital in the Morang district (2020), where low prevalence of high risk pregnancy was identified 15.81% and 14.4% respectively.^5,6^ Similarly, studies conducted in India at Puducherry (2019) and Haryana (2020) showed low prevalence of 18.3% and 12.73% respectively.^7,8^

Moreover, higher prevalence of 40.5% and 34.3% highrisk pregnancy was seen in the studies done in India at Uttarpradesh (2020) and Delhi (2023) respectively.^9,10^ The differences in prevalence could be due to difference in populations, regions, diagnostic criteria, and level of health facility.

In our study, out of the 91 (26%) high risk pregnancy cases, the most common high risk factors identified were previous history of cesarean section 25 (27.47 %), followed by hypothyroidism 19 (20.87%), gestational diabetes mellitus 15 (16.48%), malpresentation 12 (13.18%), and gestational hypertension 7 (7.69%). In the study conducted in Hyderabad, India (2020), hypertension was the most occurring disorder (53.1%) followed by younger primi and elderly gravida (33.60%).^3^ While in a hospital based study in South Western Ethiopia (2021), the leading risk factor identified in the index pregnancies were hypertension in 40 (12.5%) cases, anemia in 15 (4.8%) cases, 12 (3.8%) had Diabetes Mellitus, 9 (2.87%) had Antepartum Hemorrhage, 7 (2.2%) had malpresentation.^4^ Similarly study conducted in Shree Birendra military hospital, Nepal (2017), reported previous lower segment cesarean section as the most common identified high risk pregnancy in 34 (5.43%) cases followed by young primi gravida (3.19%), and breech (2.23%).^5^ In contrast, study done in Pakistan (2017) found that among the systemic diseases, anemia (37%) was the most common risk factor followed by hypertension (20%). Obstetrical hemorrhage (p/v bleeding) was the most prevalent complication of existing pregnancy, which contributes to the pregnancy being high risk. The percentage of teenage pregnancies was 14% and that of elderly pregnancies was 18%. However, similar to our study, the top complication of the previous pregnancy (as a risk factor) was caesarean section.^11^ In contrast study conducted in Puducherry, South India (2019), majority of the high-risk pregnancy was contributed by maternal age (teenage and elderly pregnancy) followed by pregnancy-induced hypertension (PIH).^7^ In the study conducted in Haryana, India (2020), most prevalent high-risk factors found were previous cesarean section (31.04%), anemia (31.02%), malpresentation (12.93%) and thyroid disorders (13.09%).^8^ In a study done in Karnataka, India (2017), 59.8% were having bad obstetric history, 4% were having pregnancy induced hypertension, 5.5% were elderly gravida, 3.2% were Rh negative and 22.3% were having other risk factors.^12^ A study done in a rural primary health center in Delhi, India (2023), the majority of high-risk pregnancies was caused by hypothyroidism (43.7%) followed by more than one previous LSCS (19.1%).^12^

In the current study among high risk pregnant women, 84 (92.30%) had term delivery. Most common mode of delivery was lower segment cesarean section (75.82%). Only 22 (24.18%) had vaginal delivery. High rate of caesarean section in the present study could probably due to better management of high risk pregnancy cases resulting in 100% live births. Only 13 (13.97%) babies had low-birth weight in present study. Lower prevalence of low birth weight may be partly due to majority of women were literate with middle socioeconomic status leading to improved nutritional status. In the study conducted in Hyderabad, India (2020), most of the deliveries were term deliveries (79.4%), pre term deliveries being 18.6% and post term deliveries were 2%. In the same study, 51 % deliveries were vaginal deliveries and 49% were LSCS. In contrast 31% babies had low birth weights and 5.4% were intra uterine deaths.^3^ In the study conducted in Shree Birendra military hospital, Nepal (2017), there were total 13 low birth weight baby (13.13%) similar to the present study and 2 (2%) stillbirth as fetal outcome.^5^ Study conducted in Puducherry, South India (2019), majority had spontaneous vaginal delivery (73.9%); about 10.4% gave birth to low-birth weight baby, and only 1.7% had stillbirth.^7^ In a community based study carried out in rural area of Nagpur, Maharashtra, Central India (2017) reported that among high risk pregnant women 68.06% women had caesarean section as the mode of delivery and 31.94% were normal vaginal deliveries. 20.83% were low birth weight baby with 98.61% live birth.^13^ In the study conducted in Manipal Teaching Hospital in Pokhara, Nepal (2021), cesarean deliveries were significantly more in high risk (77.9%) and extremely high risk pregnancy (89.9%) compared to low risk pregnancy (51%) with birth weight of newborns below 2500 gm was 60% in extremely high risk compared to high risk (26%) and low risk pregnancy (15%).^14^

The limitations of the study are involvement of high risk pregnant women from a single tertiary care hospital, which forms a small percentage of the population, and thereby the generalization of the results to the entire population could be a limitation. Further prospective cohort studies and randomized control trials, are warranted to provide information on high risk pregnancy related factors and pregnancy outcomes.

## CONCLUSIONS

The prevalence of high risk pregnancy in our center was found to be similar as compared to studies done in similar settings. The most common associated high risk factors were previous history of cesarean section followed by hypothyroidism, and gestational diabetes mellitus. Even though cesarean section as mode of delivery was high among high risk pregnancies, there was a good fetal outcome.
